# Genetics Behind the Glycosylation Patterns in the Biosynthesis of Dalbaheptides

**DOI:** 10.3389/fchem.2022.858708

**Published:** 2022-03-24

**Authors:** Oleksandr Yushchuk, Kseniia Zhukrovska, Francesca Berini, Victor Fedorenko, Flavia Marinelli

**Affiliations:** ^1^ Department of Biotechnology and Life Sciences, University of Insubria, Varese, Italy; ^2^ Department of Genetics and Biotechnology, Ivan Franko National University of Lviv, Lviv, Ukraine

**Keywords:** glycopeptide antibiotics, dalbaheptides, ramoplanin, teicoplanin, A40926, glycosyltransferase, biosynthetic gene cluster

## Abstract

Glycopeptide antibiotics are valuable natural metabolites endowed with different pharmacological properties, among them are dalbaheptides used to treat different infections caused by multidrug-resistant Gram-positive pathogens. Dalbaheptides are produced by soil-dwelling high G-C Gram-positive actinobacteria. Their biosynthetic pathways are encoded within large biosynthetic gene clusters. A non-ribosomally synthesized heptapeptide aglycone is the common scaffold for all dalbaheptides. Different enzymatic tailoring steps, including glycosylation, are further involved in decorating it. Glycosylation of dalbaheptides is a crucial step, conferring them specific biological activities. It is achieved by a plethora of glycosyltransferases, encoded within the corresponding biosynthetic gene clusters, able to install different sugar residues. These sugars might originate from the primary metabolism, or, alternatively, their biosynthesis might be encoded within the biosynthetic gene clusters. Already installed monosaccharides might be further enzymatically modified or work as substrates for additional glycosylation. In the current minireview, we cover recent updates concerning the genetics and enzymology behind the glycosylation of dalbaheptides, building a detailed and consecutive picture of this process and of its biological evolution. A thorough understanding of how glycosyltransferases function in dalbaheptide biosynthesis might open new ways to use them in chemo-enzymatic synthesis and/or in combinatorial biosynthesis for building novel glycosylated antibiotics.

## Introduction

Among different bacterial phyla, the mycelia-forming members of actinobacteria*‐*broadly known as actinomycetes‐remain the best antibiotic providers (Bérdy, 2005; Hutchings et al., 2019). The biosynthesis of antibiotics involves many enzymes, which are encoded by co-localized genes‐biosynthetic gene clusters (BGCs) (Medema et al., 2015). BGCs undergo modular evolution, often exchanging operons and single genes coding for biosynthetic and modification enzymes (Medema et al., 2014). Genes for glycosyltransferases (GTs) are one such example, being found in different BGCs, with corresponding proteins having relaxed substrate specificity and consequently being able to modify different natural scaffolds (Salas and Méndez, 2007).

The astonishing variability of glycosylation patterns in one group of antibiotics led to their eponymous description as glycopeptide antibiotics (GPAs, Nicolaou et al., 1999). Natural GPAs amalgamate five types of related compounds, differing in chemical structures, where, paradoxically, only types I-IV‐also known as dalbaheptides (Parenti and Cavalleri, 1989)‐are glycosylated (Nicolaou et al., 1999). All dalbaheptides possess a non-ribosomal heptapeptide aglycone differing in amino acid (aa) composition, cross-linking, and decoration (Nicolaou et al., 1999); they inhibit the growth of Gram-positive bacteria by blocking cell wall maturation (Binda et al., 2014; Yushchuk et al., 2020a). Glycosylation and acylation (a step depending on glycosylation) of dalbaheptides contribute to their antimicrobial activities, favoring dimerization and membrane localization at the site of action (Gerhard et al., 1993; Mackay et al., 1994; Beauregard et al., 1995; Snyder et al., 1998). On the other hand, excessive glycosylation does not always bring pharmacological benefits: it seemed to induce platelet aggregation in patients treated with ristocetin (Howard and Firkin, 1971; Coller and Gralnick, 1977), which was consequently withdrawn from the clinical use (Gangarosa et al., 1958).

Dalbaheptides are clinically used as drugs of last resort against multidrug-resistant Gram-positive pathogens (Marcone et al., 2018). First-generation GPAs‐vancomycin and teicoplanin (produced by different *Amycolatopsis* spp. and *Actinoplanes teichomyceticus* ATCC 31121, respectively)‐have a long and reliable history of clinical application (Jovetic et al., 2010; Binda et al., 2014; Marcone et al., 2018). In turn, natural GPAs served as precursors for three second-generation semisynthetic and clinically used GPAs (Binda et al., 2014; Butler et al., 2014): dalbavancin derived from A40926 (produced by *Nonomuraea gerenzanensis* ATCC 39727) (Crotty et al., 2016), telavancin from chloroeremomycin (from *Kibdelosporangium aridum* A82846) (Klinker and Borgert, 2015), and oritavancin from vancomycin (Crotty et al., 2016).

Although the chemical variety of glycosyl groups decorating dalbaheptide aglycones is quite remarkable (Nicolaou et al., 1999), many aspects of the genetics behind their biosynthesis and incorporation remain obscure. In this minireview, we focus on those dalbaheptides whose BGC sequences are nowadays available, and for which some experimental evidence about their glycosylation steps is reported in the literature. The model BGCs are *cep*, *bal*, *tei*, *vcm*, and *dbv*, responsible for the production of chloroeremomycin (in *K. aridum* A82846) (van Wageningen et al., 1998), balhimycin (in *Amycolatopsis balhimycina* DSM 5908) (Shawky et al., 2007), teicoplanin (Li et al., 2004), vancomycin (in *Amycolatopsis orientalis* HCCB10007) (Xu et al., 2014), and A40926 (Sosio et al., 2003), respectively. BGC from *Amycolatopsis* sp. MJM2582 (Truman et al., 2014) represents the ristocetin biosynthetic pathway and was found also in other *Amycolatopsis* spp. (Spohn et al., 2014; Liu et al., 2021). Glycosylation-related genes from more recently described BGCs for UK-68,597 (*auk* from *Actinoplanes* sp. ATCC 53533) (Yim et al., 2014a), pekiskomycin (*pek* from *Streptomyces* sp. WAC1420) (Thaker et al., 2013), keratinimicin (ker from *Amycolatopsis keratiniphila* NRRL B-24117) (Xu et al., 2019), and A50926 (from *Nonomuraea coxensis* DSM 45129) (Yushchuk et al., 2021) are also reviewed. Overall, multiple recent findings on dalbaheptide glycosylation updated the overall picture and merit a proper review, outlining what is known and why it is still worthy of further investigations.

### Delineating Steps in Dalbaheptide Glycosylation

The biosynthesis of dalbaheptides is generally divided into three distinct stages (Yim et al., 2014b, 2016; Yushchuk et al., 2020b), that is, 1) generation of non-proteinogenic aa pool, further utilized in 2) non-ribosomal biosynthesis of the oligopeptide aglycones (coupled with the oxidative cross-linking); fully cross-linked aglycones are further 3) modified in a variety of tailoring steps. All dalbaheptide BGCs encode GTs, tailoring enzymes significantly contributing to the structural variety of these antibiotics (Nicolaou et al., 1999). Non-glycosylated dalbaheptide A47934 (from *Streptomyces toyocaensis* NRRL 15009) is the only exception here; consistently, the corresponding BGC lacks GT genes (Pootoolal et al., 2002).

More in detail, different steps might be defined in the glycosylation process of dalbaheptides, layer by layer “wrapping” the aglycone. The first step includes the biosynthesis of non-conventional sugar donors for aglycone decoration. Indeed, while some dalbaheptides are decorated with sugars deriving from primary metabolism (e.g., *α*-d-mannose and *N*-acetylglucosamine (Glc*N*Ac) in teicoplanin or A40926), aglycones of vancomycin, balhimycin, and chloroeremomycin are decorated with the non-conventional monosaccharides l-vancosamine, l-4-oxovancosamine, and l-epivancosamine, respectively. In a similar manner to the biosynthesis of non-proteinogenic aa, enzymes required for the biosynthesis of such non-conventional monosaccharides are encoded within dalbaheptide BGCs. The second step (often the last one) consists of *O*-glycosylation of the aromatic aa forming the aglycone. In the third one, the installed sugars might be further modified in minor or major ways (e.g., *α*-d-mannose *O*-acetylation and Glc*N*Ac deacetylation in A40926 biosynthesis).

### Biosynthesis of Non-Conventional Monosaccharides was Required for the Glycosylation of Dalbaheptides

Conventional sugars in GPAs‐from primary metabolism‐are d-mannose, d-glucose, d-arabinose, Glc*N*Ac, and l-rhamnose. Non-conventional sugar residues include l-vancosamine, l-epivancosamine, l-4-oxovancosamine, l-ristosamine, and l-actinosamine. In addition to the aforementioned examples, l-vancosamine is present in Substitute with UK-68,597, while l-ristosamine is characteristic for ristocetin, and l-actinosamine for keratinimicin (Xu et al., 2019).

**FIGURE 1:**
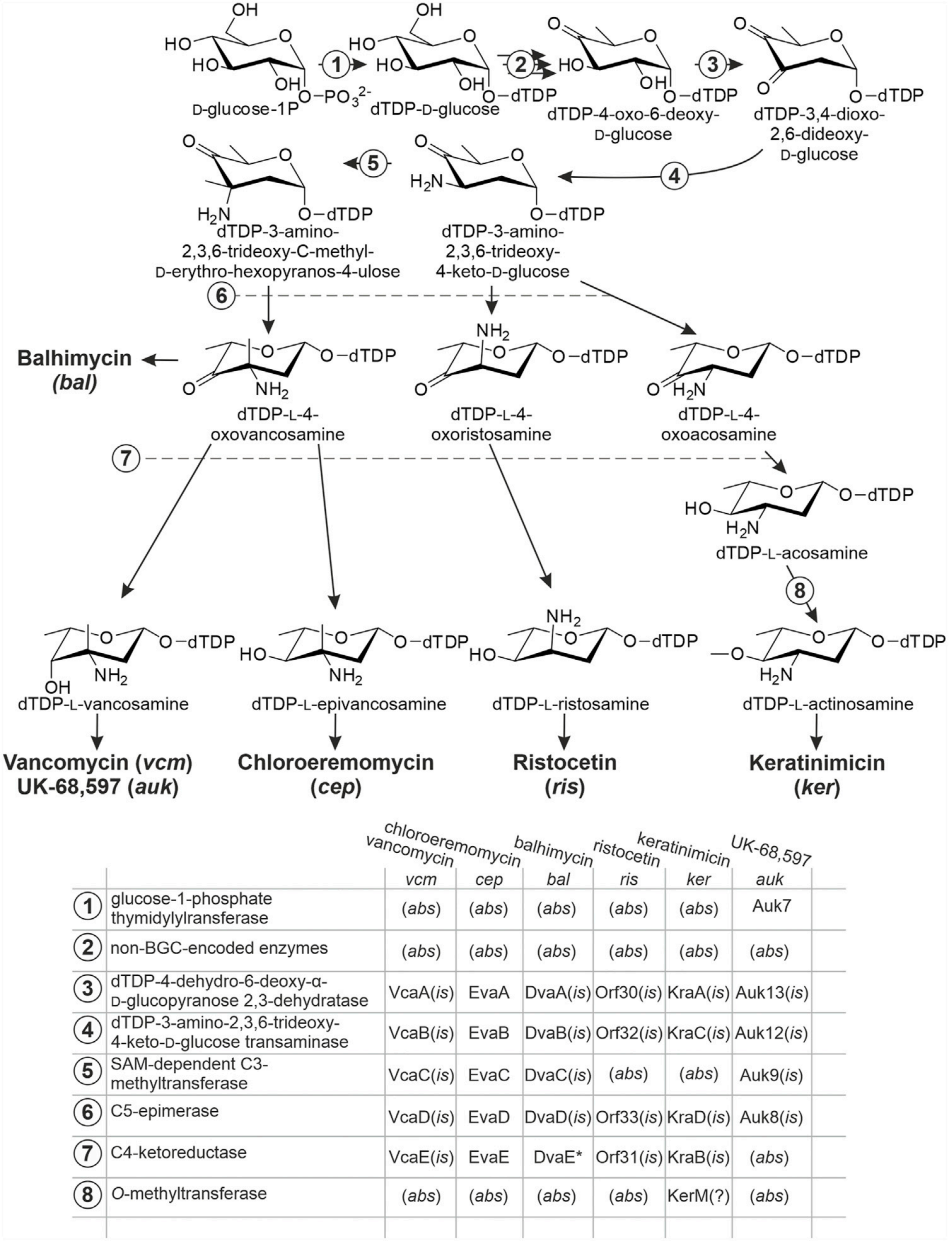
Enzymes involved in the biosynthesis of non-conventional aminosugars, decorating aglycones of some dalbaheptides. Biosynthetic pathway of l-epivancosamine serves as a model, since EvaA-B-C-D-E, coded within *cep*, are the only enzymes that were studied experimentally. Functions of all other enzymes were assigned by *in silico* comparison (*is*); (*abs*) indicates that the corresponding gene is absent from BGCs; (?) indicates that the assigned function is speculative, having no experimentally investigated prototype. Asterisk at DvaE indicates that this protein is mutated. Refer to the main text for more details.

Biosynthesis of l-epivancosamine was initially studied in chloroeremomycin producer (van Wageningen et al., 1998). *In vitro* experiments (Chen et al., 2000; Kirkpatrick et al., 2000) demonstrated how five enzymes encoded within *cep*‐namely, EvaA-E‐transformed dTDP-4-oxo-6-deoxy-d-glucose into l-epivancosamine (Figure 1). Biosynthetic routes for aminosugars decorating other dalbaheptides were deduced from this model pathway. Initially activated substrate dTDP-4-oxo-6-deoxy-d-glucose is commonly derived from d-glucose-1-phosphate by the action of non-BGC-encoded enzymes (Figure 1). One notable exception is the UK-68,597 biosynthesis, since *auk* contains a gene for glucose 1-phosphate thymidylylransferase‐*auk7* (Yim et al., 2014a)‐required for the D-glucose-1-phosphate activation. The presence of Auk7 likely positively contributes to the pool of l-vancosamine precursors in UK-68,597 biosynthesis.

*vcm* for vancomycin contains the orthologs of *evaA-E* genes, namely, *vcaA-E* (Xu et al., 2014) (Figure 1). The two sets of proteins are quite similar, sharing at least 75% of aa sequence identity (aa s.i.) in each pair (Xu et al., 2014). Nevertheless, minor differences in EvaE and VcaE seem to impact the function, making the first to convert dTDP-l-4-oxovancosamine into dTDP-l-epivancosamine, while the second yields dTDP-l-vancosamine (Figure 1). Instead, *auk* contains the orthologs of only *evaA*, *evaB*, *evaC*, and *evaD* (*auk13*, *auk12*, *auk9*, and *auk8*, respectively) (Yim et al., 2014a), lacking the ortholog of *evaE*: probably a functional homolog (or analog) of *evaE* resides outside the *auk* borders, finally contributing to l-vancosamine production. More understandable is the case of *bal* (Shawky et al., 2007), where *evaA-E* orthologs are named *dvaA-E*. Here, *evaE* ortholog *dvaE* is truncated, coding only the 99 aa C-terminal part of C4-ketoreductase (Donadio et al., 2005). Such truncated protein is non-functional, and consequently, the aminosugar biosynthesis terminates at the stage of dTDP-l-4-oxovancosamine (Figure 1). Biosynthetic pathways for l-ristosamine and l-actinosamine, coded within *ris* and *ker*, are more diverged. They both missed the EvaC ortholog (Spohn et al., 2014; Truman et al., 2014; Xu et al., 2019; Liu et al., 2021), resulting in the lack of a methyl group at the C3 position (Figure 1). Thus, biosynthesis of l-ristosamine might be attributed to the orthologs of EvaA, EvaB, EvaD, and EvaE‐Orf30, Orf32, Orf33, and Orf31, respectively (Truman et al., 2014). The same protein set is encoded within *ker*‐KraA-D (Xu et al., 2019). The major difference between l-actinosamine and l-ristosamine is *O*-methylation of the C4-position. It is still unknown how this methylation is achieved; however, the annotation of *ker* reveals the presence of a gene coding for an *O-*methyltransferase‐*kerM* (Xu et al., 2019)*.* Since the aglycone of keratinimicin lacks any *O*-methylations, it seems plausible that KerM catalyzes the ultimate step of l-actinosamine biosynthesis (Figure 1).

In all cases described above the orthologs of *evaA-E-B-D* are most likely co-expressed, forming one operon, while the orthologs of *evaC* belong to a separate transcriptional unit (Shawky et al., 2007; Liu et al., 2021). *auk* is an exception, with *auk8-9* (*evaD-C*) and *auk12-13* (*evaA-B*) probably belonging to different operons (Yim et al., 2014a).

### GTs Involved in Dalbaheptide Glycosylation

All GTs decorating aglycones of dalbaheptides belong to two families, according to the Carbohydrate-Active enZYmes Database (CAZy, http://www.cazy.org, Drula et al., 2022). GTs responsible for the installation of non-conventional aminosugars and conventional d-glucose, d-arabinose, Glc*N*Ac, and l-rhamnose, belong to the GT1 family. These GTs require sugar substrates to be either dTDP- or UDP-activated, and share a unique two-domain structure (the so-called GT-B fold, Lairson et al., 2008), having C- and N-terminal Rossmann-like domains connected by a flexible linker region (Zhang et al., 2020). Recognition sites for the donor NDP-activated sugars are located at C-terminal domains (Chang et al., 2011), while N-terminal domains contain the acceptor binding site for dalbaheptide aglycone (Chang et al., 2011; Zhang et al., 2020). GTs of the second‐GT39‐family are responsible for the installation of d-mannose and require d-mannosyl-1-phosphoundecaprenol as a donor substrate. Large hydrophobic GT39-GTs are predicted as membrane-associated, having a GT-C fold with multiple transmembrane helices and intracellular active sites (Lairson et al., 2008).

In the aforementioned dalbaheptides, GT1-GTs attach sugar residues preferentially at AA-4 (4-hydroxyphenylglycine, Hpg) and AA-6 (*β*-hydroxytyrosine, Bht) of the aglycone or add additional monosaccharides to already existing mono/di/trisaccharides at AA-4. Fully cross-linked aglycones serve as acceptor substrates for GT1-GTs under physiological conditions, albeit some GT1-GTs were able to recognize partially cross-linked aglycones under certain experimental conditions, for instance, in mutasynthesis approaches (Weist et al., 2004; Butz et al., 2008). GT39-GTs attach mannose at AA-7 (3,5-dihydroxyphenylglycine, Dpg). The presence of multiple GTs within one pathway might result in the production of mixtures of related congeners, differing in glycosylation patterns. Dalbaheptides glycosylated at other AA positions were also described (Nicolaou et al., 1999), indicating that glycosylation might also occur at 1) AA-2 (Bht) and AA-1 (Hpg) in type II aglycone, and 2) AA-1 (Hpg) and AA-3 (Bht) in type III aglycone. Unfortunately, we currently lack any genomic information on the producers of these molecules, which would merit further investigations.

**FIGURE 2:**
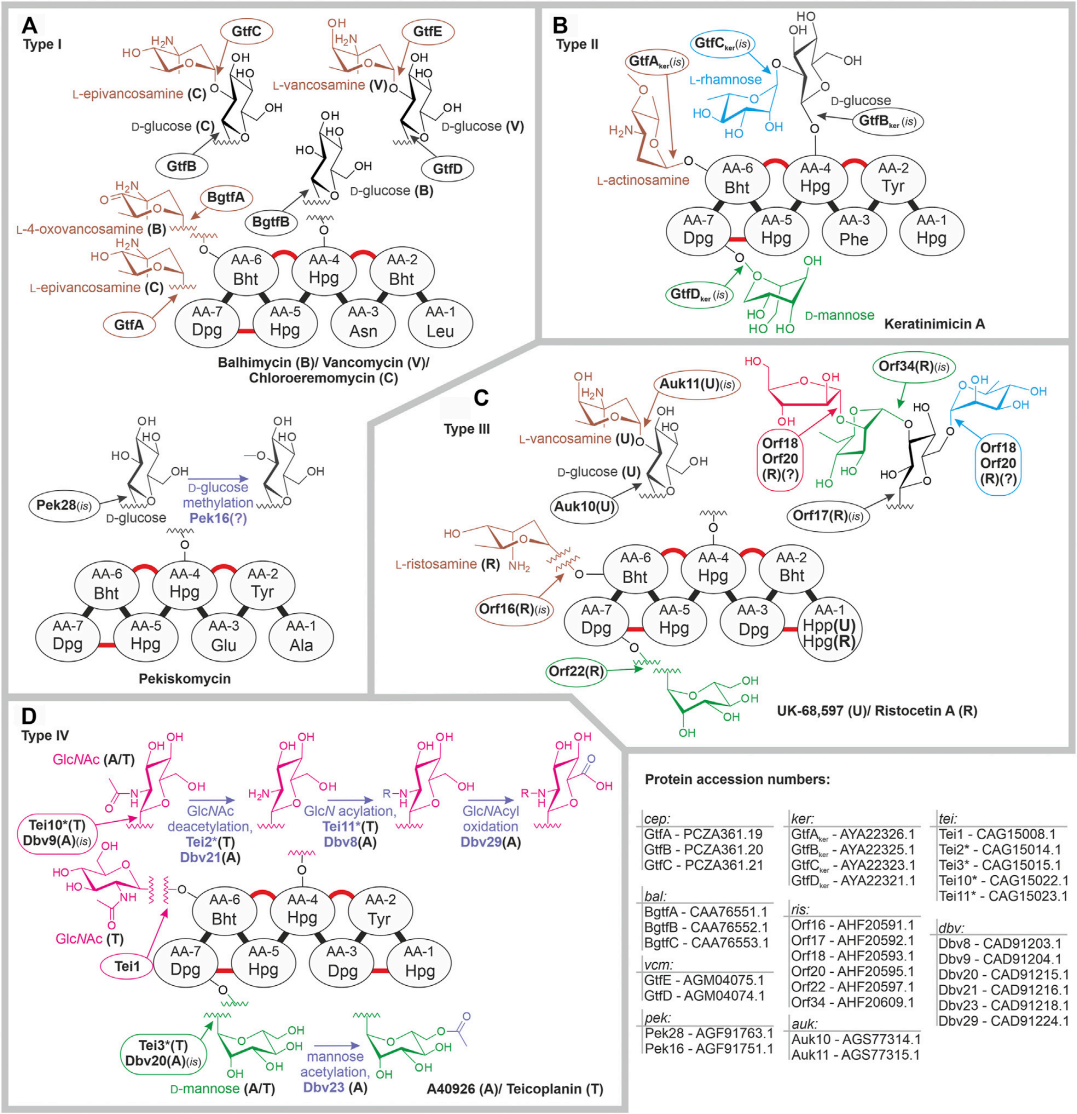
Glycosylation patterns of type I **(A)**, type II **(B)**, type III **(C)**, and type IV **(D)** dalbaheptides and enzymes involved therein. Refer to the main text for more details. Dalbaheptide aglycones are depicted schematically with cross-links shown in red, and chlorination and sulfation sites are not shown. Aglycone amino acid abbreviations mean following: Leu‐leucine; Asn‐asparagine; Ala‐alanine; Glu‐glutamine; Phe‐phenylalanine; Tyr‐tyrosine; Bht‐*ß*-hydroxytyrosine; Dpg‐3,5-dihydroxyphenylglycine; Hpg‐4-hydroxyphenylglycine; Hpp‐4-hydroxyphenypyruvate; Tyr‐. Glc*N* stated for *N-*glucosamine, GlcNAcyl for N-acylglucosamine. All enzymes, whose functions were assigned by *in silico* comparison are marked with (*is*); (?) indicates that function was assigned *in silico* without experimentally investigated prototype or exact function remains unknown. For fast access to protein sequences mentioned in this figure, use following links: *cep*: GtfA‐PCZA361.19, GtfB‐PCZA361.20, GtfC‐PCZA361.21; *bal*: BgtfA‐CAA76551.1, BgtfB‐CAA76552.1, BgtfC‐CAA76553.1; *vcm*: GtfE‐AGM04075.1, GtfD‐AGM04074.1; *pek*: Pek28‐AGF91763.1, Pek16‐AGF91751.1; *ker*: GtfA_ker_‐AYA22326.1, GtfB_ker_‐AYA22325.1, GtfC_ker_‐AYA22323.1, GtfD_ker_‐AYA22321.1; *ris*: Orf16‐AHF20591.1, Orf17‐AHF20592.1, Orf18‐AHF20593.1, Orf20‐AHF20595.1; Orf22‐AHF20597.1, Orf34‐AHF20609.1; *auk*: Auk10‐AGS77314.1, Auk11‐AGS77315.1; *tei*: Tei1‐CAG15008.1, Tei2*‐CAG15014.1, Tei3*‐CAG15015.1, Tei10*‐CAG15022.1, Tei11*‐CAG15023.1; *dbv*: Dbv8‐CAD91203.1, Dbv9‐CAD91204.1, Dbv20‐CAD91215.1, Dbv21‐CAD91216.1, Dbv23‐CAD91218.1, Dbv29‐CAD91224.1.

Type I dalbaheptides chloroeremomycin, balhimycin, and vancomycin served as the first models for experimental investigation of GT functions. Corresponding BGCs encode slightly different sets of GT1-GTs: GtfA, GtfB, and GtfC in *cem*; BgtfA, BgtfB, and BgtfC in *bal*; and GtfD and GtfE in *vcm.* Orthologous GTs GtfA and BgtfA install l-epivancosamine and l-4-oxovancosamine at AA-6 of chloroeremomycin and balhimycin, respectively. *vcm* does not encode GtfA ortholog, explaining why vancomycin is not glycosylated at AA-6. Peculiarly, this particular difference between vancomycin and chloroeremomycin seems to augment the antimicrobial activity of the latter, implying that l-epivancosamine at AA-6 facilitates cell wall binding (Nagarajan, 1993; Allen et al., 2002). GtfB, BgtfB, and GtfE are orthologs, glucosylating AA-4 in the biosynthesis of all three antibiotics (Figure 2A, Pelzer et al., 1999; Losey et al., 2001; Mulichak et al., 2001). Then, GtfC and GtfD attach l-epivancosamine or l-vancosamine to d-glucose at AA-4 in the biosynthesis of chloroeremomycin and vancomycin, respectively (Figure 2A, Losey et al., 2001, 2002; Mulichak et al., 2004). GtfC, GtfD, and BgtfC are orthologous proteins, but balhimycin lacks a disaccharide at AA-4, which could be found only in balhimycin V (a congener produced in residual amounts) (Pelzer et al., 1999; Stegmann et al., 2010). This might be due to the low affinity of BgtfC for the donor substrate‐l-4-oxovancosamine, produced as a consequence of DvaE mutation. More recently described type I dalbaheptide pekiskomycin is only glucosylated at AA-4, coherently with the only 1 GT encoded in *pek*: Pek28, which is a GtfB ortholog (Figure 2A) (Thaker et al., 2013).

So far, little is known about GTs decorating aglycones of type II dalbaheptides. *ker* (single type II BGC sequenced) carries three genes for GT1-GTs and one gene for GT39-GT (Xu et al., 2019). Among *ker-*encoded GT1-GTs, *gtfA*_*ker*_, *gtfB*_*ker*_, and *gtfC*_*ker*_ (*ker* was added to distinguish them from *cep* genes) are orthologs of *gtfA*, *gtfB*, and *gtfC*, respectively (Xu et al., 2019). Thus, GtfB_ker_ most likely installs d-glucose at AA-4, and l-rhamnose is then appended to d-glucose by GtfC_ker_; this leaves GtfA_ker_ responsible for the attachment of l-actinosamine at AA-6 (Figure 2B). Single *ker-*encoded GT39-GT GtfD_ker_ most probably attaches d-mannose at AA-7 (Figure 2B).

More information is available on the GTs involved in the biosynthesis of type III dalbaheptides. Ristocetin BGC encodes 6 GTs, four of GT1 family and two belonging to the GT39 family (Truman et al., 2014). Phylogenetic reconstruction allowed to assign functions to 4 GTs, assuming that Orf16 attaches l-ristosamine at AA-6, Orf17‐d-glucose at AA-4, then appended with d-mannose by Orf34; finally, a second mannosyltransferase‐Orf22‐was expected to act at AA-7 (Figure 2C, Truman et al., 2014), as later confirmed by its heterologous expression in *Streptomyces coelicolor* carrying *sta* (Yim et al., 2016)*.* Functions of Orf18 and Orf20 were not assigned, since these proteins were distantly related to known GTs (Truman et al., 2014). Another type III dalbaheptide‐UK-68,597‐is decorated with 2-L-vancosaminyl-d-glucose disaccharide at AA-4 (Yim et al., 2014a), while *auk* carries three genes for GT1-GTs. *In vitro* assay showed that Auk10 (GtfB ortholog) is responsible for glucosylation; *in silico* analysis then suggested l-vancosamine to be installed by Auk11 (GtfC ortholog, Figure 2C) (Yim et al., 2014a). Although the third GT, Auk14, is a GtfA ortholog, UK-68,597 lacks sugar residues at AA-6. *In vitro* assay suggested that Auk14 might be inactive or possess a very low affinity to substrates available in UK-68,597 biosynthesis, as observed with BgtfA (Yim et al., 2014a).

Glycosylation events that take place in type IV dalbaheptide biosynthesis are well defined (Figure 2D). *tei* and *dbv* BGCs encode three and two GTs, respectively. Accordingly, both antibiotics carry a Glc*N*Ac moiety at AA-4 and d-mannose at AA-7, while teicoplanin aglycone is also decorated with another Glc*N*Ac at AA-6. GtfB ortholog‐Tei10*‐installs the Glc*N*Ac moiety at AA-4, as demonstrated from multiple *in vivo* and *in vitro* experiments (Li et al., 2004; Howard-Jones et al., 2007; Truman et al., 2009; Yushchuk et al., 2016, 2020b). Consistently, Dbv9‐a Tei10* ortholog‐is supposed to play the same role in A40926 glycosylation. Tei1 was shown to attach Glc*N*Ac to teicoplanin aglycone at AA-6 (Li et al., 2004), whereas *dbv* lacks any Tei1 ortholog, explaining why A40926 has no sugars at AA-6. Finally, Tei3* was shown to be responsible for the decoration of teicoplanin aglycone with d-mannose at AA-7 (Yushchuk et al., 2016), implying that Dbv20 (Tei3* ortholog) has the same function in A40926 biosynthesis (Figure 2D). Interestingly, ramoplanin BGC (*ramo*), recently shown to be genetically related to *tei* (Waglechner et al., 2019), encodes a homolog of Tei3*‐Ramo29 (45% aa s.i., Chen et al., 2013). Ramoplanin is a clinically relevant peptide antibiotic produced by *Actinoplanes ramoplaninifer* ATCC 33076 (Marcone et al., 2017). Unlike dalbaheptides, it carries a 4-d-mannosyl-d-mannose disaccharide, instead of a single d-mannose residue. Ramo29 was shown to install the first d-mannose residue (Chen et al., 2013), but the GT responsible for the second mannosylation remains unknown. This merits further investigation, since such GT looks like a promising tool to further modify mannosylated dalbaheptides.

Concluding this section, it is interesting to report that genes for GTs tend to form one operon in type I-III BGCs (Shawky et al., 2007; Liu et al., 2021), being more scattered in type IV BGCs (Alduina et al., 2007; Yushchuk et al., 2019). It is also notable that genes for mannosyltransferases are present in different types of BGCs coming from distant actinobacterial lineages. This might indicate d-mannose residues at AA-7 to be an ancestral feature for all dalbaheptides.

### Further Modification Occurring on Attached Sugar Residues

Some further modifications of attached sugars occur during the biosynthesis of dalbaheptides, although they are quite rare. The first example comes from pekiskomycin, having d-glucose methylated. *pek* encodes two methyltransferases. One of them‐Pek30‐was experimentally shown to methylate the N-terminus of A47934 aglycone (Yim et al., 2016), leaving the other‐Pek16‐possibly responsible for d-glucose methylation (Figure 2A). Another notable modification is the acylation of AA-4 Glc*N*Ac in type IV dalbaheptides, such as teicoplanin and A40926. To achieve this modification, Glc*N*Ac at AA-4 is first deacetylated with orthologous deacetylases Tei2*/Dbv21 (Ho et al., 2006; Truman et al., 2006), and *N*-glucosamine is then acylated with orthologous acyltransferases Tei11*/Dbv8 (Figure 2D) (Li et al., 2004; Kruger et al., 2005; Howard-Jones et al., 2007; Yushchuk et al., 2016). Peculiarly, orthologs of Tei2*/Dbv21 are present in many (if not in all) BGCs for non-acylated type I-III dalbaheptides. The one from *cep*‐CepI‐was studied *in vitro* and shown to be inactive due to a single aa substitution (Truman et al., 2008). The omnipresence of tei2* orthologs in type I-III BGCs induces to speculate that the *N-*acylglucosamine moiety at AA-4 is an ancestral feature, lost or modified in many evolutionary lineages of dalbaheptides.

Modifications of A40926 sugars do not end with acylation. *N*-acylglucosamine moiety is further oxidized to *N*-acylaminoglucuronic acid group by Dbv29 (Figure 2D) (Li et al., 2007). The biological role of such modification is unclear, although it seems to reduce the A40926 antimicrobial activity (Malabarba et al., 1995). Peculiarly, *noc* BGC in *N. coxensis* DSM 45129 lacks an ortholog for *dbv29*, coding the biosynthesis of non-oxidized A40926 analog‐dalbaheptide A50926 (Yushchuk et al., 2021). Finally, the d-mannose residue at AA-7 of A40926 is *O-*acetylated with Dbv23 (Figure 2D) (Sosio et al., 2010). This modification is unstable and fades away in the alkaline extraction of A40926 (Alt et al., 2019); once again, its biological role is unclear, although it might be important for the regulation of antibiotic export and self-resistance (Alduina et al., 2020).

## Conclusion and Outlook

Further research of dalbaheptide glycosylation is important for several reasons. Understanding of GTs donor- (activated sugar) and acceptor- (aglycone) substrate specificities will allow further chemical derivatization of these scaffolds using *in vitro* chemo-enzymatic synthesis (Nakayama et al., 2014) or *in vivo* combinatorial biosynthesis (Yim et al., 2016). While the first approach has been widely investigated in the past for generating novel hybrid GPAs by combining natural and synthetic aglycones and sugars (as reviewed in Marcone et al., 2018), *in vivo* combinatorial biosynthesis is promising, but still rather limited in its applications. Alternatively, the two-domain architecture of GT1-GTs might be exploited to create “chimaeras” with an expanded functional repertoire (Truman et al., 2009). Finally, a better comprehension of glycosylation mechanisms will contribute to tracing out a more complete picture of dalbaheptide evolution. Unlike other aspects of glycopeptide biosynthesis (Donadio et al., 2005; Waglechner et al., 2019; Andreo-Vidal et al., 2021), phylogenomics of GTs and sugar modification enzymes has not been studied yet. We believe that such reconstruction might open new scenarios on the evolution of antibiotic biosynthetic pathways.
